# Comparative performance of PD‐L1 scoring by pathologists and AI algorithms

**DOI:** 10.1111/his.15432

**Published:** 2025-02-17

**Authors:** Markus Plass, Gheorghe‐Emilian Olteanu, Sanja Dacic, Izidor Kern, Martin Zacharias, Helmut Popper, Junya Fukuoka, Sosuke Ishijima, Michaela Kargl, Christoph Murauer, Lipika Kalson, Luka Brcic

**Affiliations:** ^1^ Diagnostic and Research Institute of Pathology Medical University of Graz Graz Austria; ^2^ Department of Pathology British Columbia Cancer Agency Vancouver BC Canada; ^3^ Department of Pathology and Laboratory Medicine University of British Columbia Vancouver BC Canada; ^4^ University of Pittsburgh Medical Center Pittsburgh PA USA; ^5^ Cytology and Pathology Laboratory University Clinic of Respiratory and Allergic Diseases Golnik Slovenia; ^6^ Department of Pathology Informatics Nagasaki University Graduate School of Biomedical Sciences Nagasaki Japan; ^7^ Department of Pathology Kameda Medical Center Kamogawa Japan; ^8^ Present address: Department of Pathology Yale School of Medicine New Haven CT USA

**Keywords:** AI algorithms, NSCLC, pathologists, PD‐L1 expression, tumour proportion score (TPS)

## Abstract

**Aim:**

This study evaluates the comparative effectiveness of pathologists versus artificial intelligence (AI) algorithms in scoring PD‐L1 expression in non‐small cell lung carcinoma (NSCLC). Immune‐checkpoint inhibitors have revolutionized NSCLC treatment, with PD‐L1 expression, measured as the tumour proportion score (TPS), serving as a critical predictive biomarker for therapeutic response.

**Methods and Results:**

In our analysis, 51 SP263‐stained NSCLC cases were scored by six pathologists using light microscopy and whole‐slide images (WSI), alongside evaluations by two commercially available software tools: uPath software (Roche) and the PD‐L1 Lung Cancer TME application (Visiopharm). The study examined intra‐ and interobserver agreement among pathologists at TPS cutoffs of 1% and 50%, revealing moderate interobserver agreement (Fleiss' kappa 0.558) for TPS <1% and almost perfect agreement (Fleiss' kappa 0.873) for TPS ≥50%. Intraobserver consistency was high, with Cohen's kappa ranging from 0.726 to 1.0. Comparisons between the AI algorithms and the median pathologist scores showed fair agreement for uPath (Fleiss' kappa 0.354) and substantial agreement for the Visiopharm application (Fleiss' kappa 0.672) at the 50% TPS cutoff.

**Conclusion:**

These results indicate that while there is strong interobserver concordance among pathologists at higher TPS levels, the performance of AI algorithms is less consistent. The study underscores the need for further refinement of AI tools to match the reliability of expert human evaluation, particularly in critical clinical decision‐making contexts.

## Graphical abstract

Pathologists demonstrate higher consistency than analysed AI algorithms in PD‐L1 scoring at critical Tumour Proportion Score cutoffs in non‐small cell lung cancer. This emphasizes the need to enhance AI tools to better support pathologists in everyday assessments.

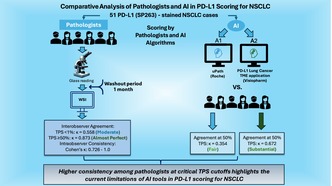

**Abbreviations**
AI: artificial intelligence
AUC: area under the curve
CAP‐PLQC: College of American Pathologists Pathology and Laboratory Quality Center
CI: confidence interval
DL: deep learning
DP: digital pathology
FDA: Food and Drug Administration
FFPE: formalin‐fixed paraffin‐embedded
ICC: intraclass correlation coefficient
ICI: immune checkpoint inhibitor
IHC: immunohistochemistry
NSCLC: non‐small cell lung cancer
PD‐L1: programmed death‐ligand 1
ROI: region of interest
RUO: research‐use‐only
TPS: tumor proportion score
WSI: whole slide image

## Introduction

Since the US Food and Drug Administration's (FDA) approval of the first immunotherapy for lung cancer in 2015, immunotherapy has become one of the main treatment options for NSCLC, improving patient survival.^1^, ^2^ Programmed death‐ligand 1 (PD‐L1), an immune checkpoint protein found on resident macrophages, immune, and tumour cells, is crucial in evaluating non‐small cell lung cancer (NSCLC) cases for immune checkpoint inhibitor (ICI) therapy. With multiple immunohistochemistry (IHC) antibody clones available (e.g. 28–8, 22C3, SP263, and SP142) and various assessment methods, there is notable inter‐ and intraobserver variability in PD‐L1 scoring, highlighting the potential role for deep learning (DL) models in enhancing accuracy.^3^, ^4^ The PD‐L1 expression by IHC has been implemented as a standard predictive biomarker of response to anti‐PD‐1/PD‐L1 immunotherapy.^5^ The tumour proportion score (TPS), the percentage of positive tumour cells, is the recommended criterion of PD‐L1 expression in NSCLC in clinical practice. For the first‐line and second‐line treatment of NSCLC with approved PD‐1/PD‐L1 inhibitors, different IHC assays with different regulatory designations and cutoff values for the TPS have been approved in different countries/regions.^5^ TPS cutoff points relevant for therapy decisions in NSCLC are TPS ≥1% and TPS ≥50%.^5^ Integrating artificial intelligence (AI) and digital pathology (DP) in diagnostic medicine, particularly in PD‐L1 evaluation in lung cancer treatment, represents a pivotal advance.^6^, ^7^ AI algorithms are a significant innovation that addresses traditional challenges in pathological assessments, such as labour‐intensiveness and subjectivity in PD‐L1 scoring.^8^ AI's capacity for rapid processing of extensive datasets accelerates the diagnostic process, which is crucial in cancer care, where timely intervention is critical.^6^ Furthermore, AI's precision in molecular quantification, notably in PD‐L1 marker analysis, ensures consistent and reliable scoring, essential for informed therapeutic decisions. AI's objectivity, offering unbiased evaluations, contrasts with the potential interobserver variability and subjective interpretations inherent in human assessments.^9^, ^10^, ^11^ This uniformity is vital for consistent treatment outcomes. Additionally, AI's ability to discern complex patterns in tissue samples enhances diagnostic accuracy, especially in ambiguous or borderline PD‐L1 scoring cases, leading to more accurate and tailored treatment strategies. AI augments pathologists' capabilities, allowing them to focus on more complex diagnostic tasks.^12^, ^13^, ^14^, ^15^ Synergy between AI and human expertise fosters a more advanced, accurate diagnostic process in pathology. In immuno‐oncology, precise PD‐L1 expression evaluation in NSCLC is crucial for guiding therapy, particularly for PD‐1/PD‐L1 inhibitor treatments.^16^, ^17^ Traditional assessment methods, largely dependent on pathologists' interpretations, often show variability, affecting the consistency and reliability of PD‐L1 scoring. This variability can significantly influence clinical decisions, especially in borderline cases. Advances in DP and AI aim to enhance the accuracy of PD‐L1 expression analysis, addressing the subjectivity of manual evaluations.^16^, ^17^ While AI algorithms hold considerable potential for enhancing the assessment of PD‐L1 expression in NSCLC, existing research indicates that AI cannot yet fully replace human pathologists.^18^, ^19^ A key challenge lies in the variability of concordance observed between AI models and human assessments. This variability necessitates ongoing human oversight to ensure accurate and reliable PD‐L1 scoring in clinical practice. For instance, a study published by van Eekelen *et al*.^18^ demonstrated a strong correlation between AI‐powered and manual scoring of PD‐L1, with correlation coefficients ranging from 0.73 to 0.85. However, discrepancies were noted, particularly at lower PD‐L1 expression thresholds, where AI systems tended to identify a higher prevalence of PD‐L1–positive cases compared to human pathologists.^18^ This tendency towards overestimation by AI algorithms in specific scenarios could have significant implications for treatment decisions, potentially leading to inappropriate patient selection for immunotherapy. Therefore, human validation remains crucial to mitigate the risk of inaccurate PD‐L1 scoring and optimize patient care. Building upon previous research, our study sought to assess intra‐ and interobserver variability in PD‐L1 TPS interpretation by pathologists using light microscopy and digitalized whole‐slide images (WSI). We compared these interpretations with results from two commercial PD‐L1 analysis algorithms.

## Materials and methods

### Study cohort

**Figure 1:**
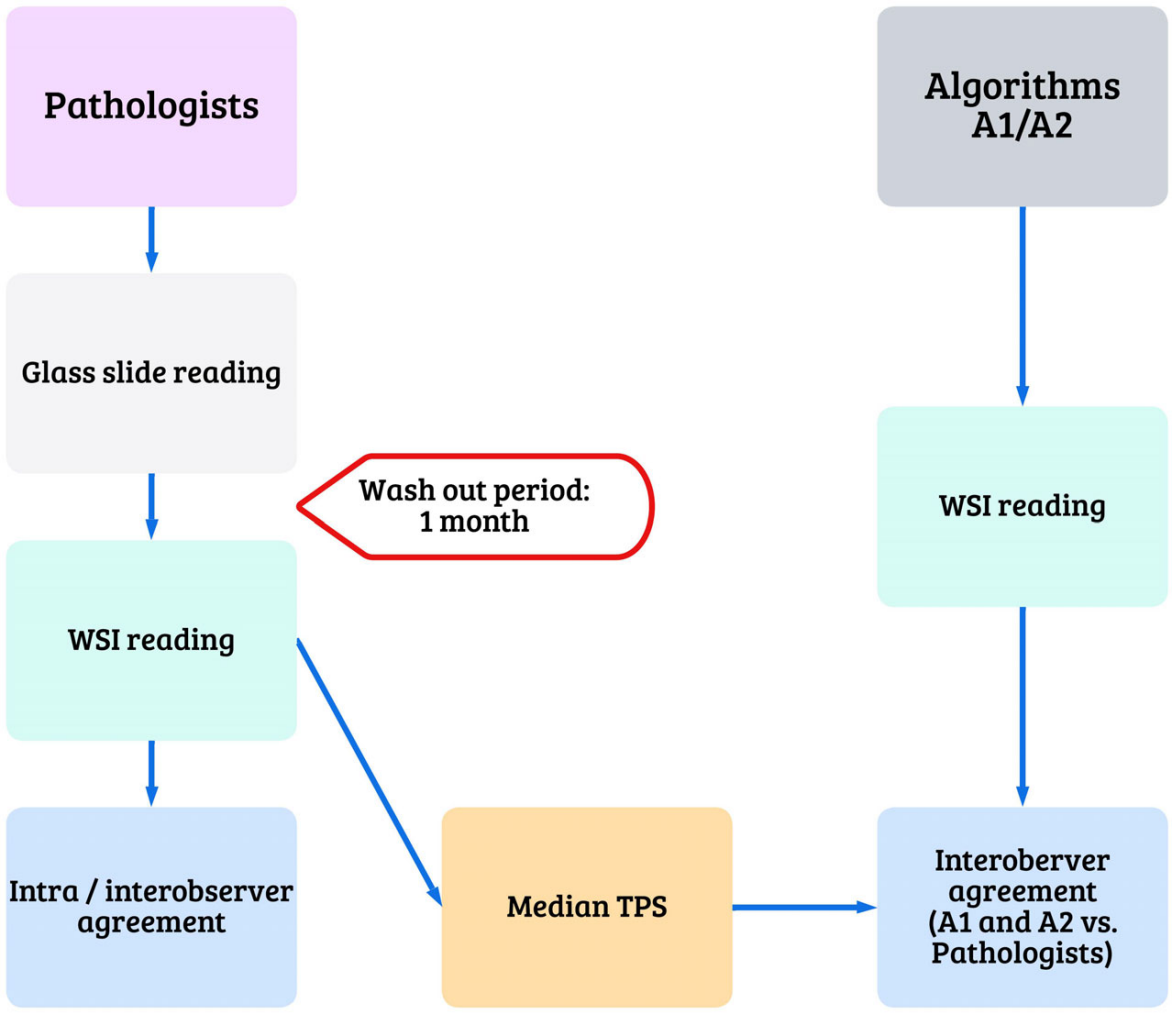
Design outline describing the flow of the methodology used in the study. Whole‐slide imaging (WSI), Tumour proportion score (TPS). [Color figure can be viewed at wileyonlinelibrary.com]

Formalin‐fixed paraffin‐embedded (FFPE) samples of 51 consecutive patients diagnosed with NSCLC (34 adenocarcinomas and 17 squamous cell carcinomas) in 2020, at the Diagnostic and Research Institute of Pathology, Medical University of Graz were included in this retrospective study. Of the 51 samples, 26 were bronchoscopy biopsies, and 25 were surgical resections. See study design outline in Figure 1.

This retrospective study conformed to the principles outlined in the Declaration of Helsinki (as revised in 2013). It was approved by the Ethics Committee of the Medical University of Graz (30‐105 ex 17/18).

### Immunohistochemistry

Haematoxylin–eosin (H&E)‐stained slides were reevaluated to confirm the presence of a minimum of 100 tumour cells. The analysis of the tumour tissue with antibodies against PD‐L1 was performed on freshly cut 4‐μm‐thick paraffin sections. VENTANA PD‐L1 (clone SP263, ready‐to‐use) Assay (Ventana Medical Systems, a member of Roche Group, Tucson, AZ, USA) was applied according to the manufacturer's protocol on the BenchMark ULTRA platform (Ventana Medical Systems), with the usage of adequate positive and negative controls.

### Whole‐slide images

Matched H&E‐stained slides and PD‐L1‐stained slides were scanned with a resolution of 0.25 μm/pixel on the PANORAMIC1000 slide scanner (3DHISTECH, Budapest, Hungary). Digital images created by the PANORAMIC1000 slide scanner were uploaded to the CaseCenter (3DHISTECH) hosted by the Medical University Graz for scoring by pathologists. These WSIs were also used by Visiopharm's PD‐L1 Lung Cancer TME application (Visiopharm, Hoersholm, Denmark).

In addition, the PD‐L1‐stained slides were also scanned on the Ventana DP200 slide scanner (Roche Diagnostics International, Rotkreuz, Switzerland). The uPath software (Roche Diagnostics) was used to manage and view the WSIs created by the Ventana DP200 slide scanner.

### Scoring of PD‐L1

#### Scoring by pathologists

The evaluation of PD‐L1 staining was performed only on tumour cells. Any intensity of either partial or complete membranous staining was regarded as positive. The percentage of positively stained tumour cells was recorded as follows: 0%, 1%, 5%, 10%, and up to 100% in 10% increments. The evaluation was performed by six pathologists (five pulmonary pathologists, and one pathologist in training). First, pathologists scored glass slides, and after a washout period of at least 1 month (as recommended by CAP‐PLQC guidelines),^20^ all pathologists reviewed WSIs of the same cases.

#### Scoring by AI algorithms

Two different AI algorithms for PD‐L1 TPS scoring were applied to the same cases. Algorithm 1 (A1, uPath PD‐L1 (SP263) image analysis software for PD‐L1 evaluation; Roche), certified as an *in vitro* diagnostics device compliant with the European In‐Vitro Diagnostic Devices Directive (IVDD 98/79/EC) for classification of PD L1 TPS ≥50%. As required, it was applied to the WSIs scanned on the Ventana DP200 slide scanner (Roche). When applying A1 on a WSI, the tumour area had to be selected manually by the pathologist.

Algorithm 2 (A2, PD‐L1 Lung Cancer TME, Visiopharm) was applied to the WSIs scanned on the PANORAMIC1000 slide scanner (3D Histech). A2 is fully automated and does not require manual annotation of the region of interest.

### Statistical analyses

For statistical analysis the statistics software IBM SPSS® 27.0.0 (IBM, Armonk, NY, USA) was used.

#### Data

The PD‐L1 TPS percentage results of pathologists and algorithms were transformed into categorical data comprising category 1: PD‐L1 TPS <1%, category 2: 1% ≥PD‐L1 TPS <50%, and category 3: PD‐L1 TPS ≥50.

In addition, the PD‐L1 TPS percentage results were also transformed into dichotomous data at the TPS 50% cutoff (PD‐L1 TPS <50% and PD‐L1 TPS ≥50%).

#### Agreement measures

Raw agreement indices, i.e. overall agreement and specific agreement for each of the three categories, were calculated for the assessment of intraobserver agreement between a pathologist's PD‐L1 TPS categorical results obtained by glass slide reading versus WSI reading.

The intraclass correlation coefficient (ICC) two‐way mixed‐effects, absolute agreement, and single measurement^21^ was calculated for estimating intraobserver agreement between a pathologist's PD‐L1 TPS results (expressed as percentage values) obtained by glass slide reading versus WSI reading. ICC values <0.5, between 0.5 and 0.75, between 0.75 and 0.9, and >0.9 can be interpreted as poor, moderate, good, and excellent agreement, respectively.^22^

Cohen's kappa was calculated for estimating intraobserver agreement between a pathologist's PD‐L1 TPS categorical results obtained by glass slide reading versus WSI reading. Cohen's kappa values were interpreted as follows: 0–0.20 “no agreement”, 0.21–0.39 “minimal agreement”, 0.40–0.59 “weak agreement”, 0.60–0.79 “moderate agreement”, 0.80–0.90 “strong agreement”, >0.90 “almost perfect agreement”.^23^

Fleiss' kappa was calculated to estimate interobserver agreement among the participating six pathologists' PD‐L1 TPS categorical results obtained by WSI reading and glass slide reading. Fleiss´ kappa values were interpreted as follows: 0.01–0.20 “slight agreement”, 0.21–0.40 “fair agreement”, 0.41–0.60 “moderate agreement”, 0.61–0.80 “substantial agreement”, and 0.81–1.00 “almost perfect agreement”.

Furthermore, Fleiss' kappa was also used for the assessment of interobserver agreement between pathologists and algorithms for categorical data and for dichotomous data at the TPS 50% cutoff, since A1 is certified for this cutoff. Here the results of automatic image analysis algorithms were compared with the median result of the six participating pathologists.

All estimation results of the analysis are expressed as mean and asymptotic 95% confidence interval (CI). The values *P* < 0.05 were considered statistically significant.

## Results

### Intraobserver agreement for PD‐L1 TPS scoring of glass slides versus WSI

**Figure 2:**
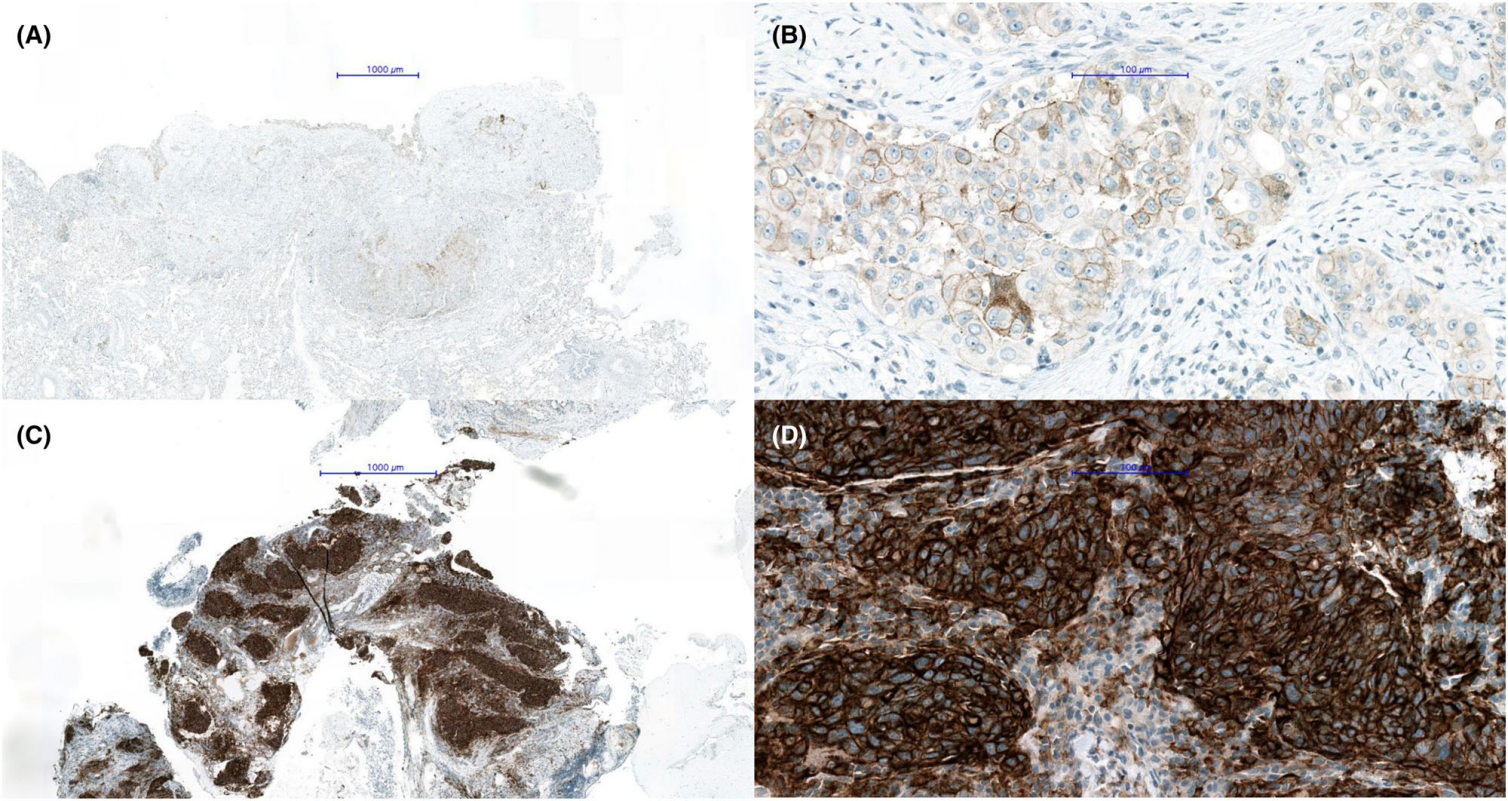
Examples of WSI readings of PD‐L1 IHC. Low and high magnification. For (**A**) and (**B**) pathologists' PD‐L1 TPS scores were >50%, whereas both algorithms (A1 and A2) reported TPS scores <50% (**C**) and (**D**) complete agreement was observed, with PD‐L1 TPS scores >50% as determined by the pathologists and both algorithms (A1 and A2). [Color figure can be viewed at wileyonlinelibrary.com]

**Figure 3:**
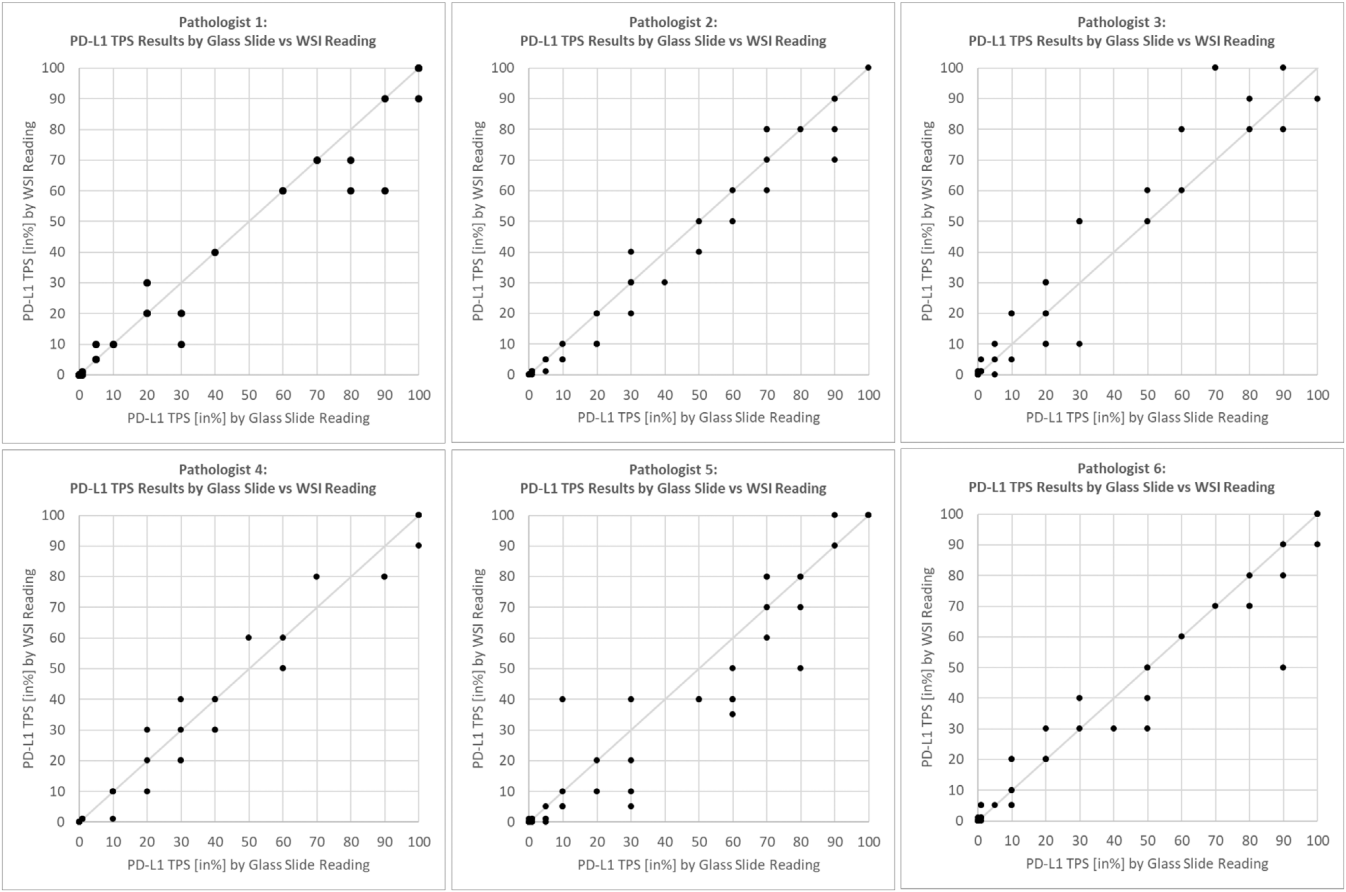
Pathologist's PD‐L1 TPS % results by glass slide versus WSI reading. Each of the six diagrams shows a specific participating pathologist's PD‐L1 TPS percentage results for glass slide reading (horizontal axis) versus WSI reading (vertical axis). For perfect intraobserver agreement, all points would lie on the diagonal line.

**Figure 4:**
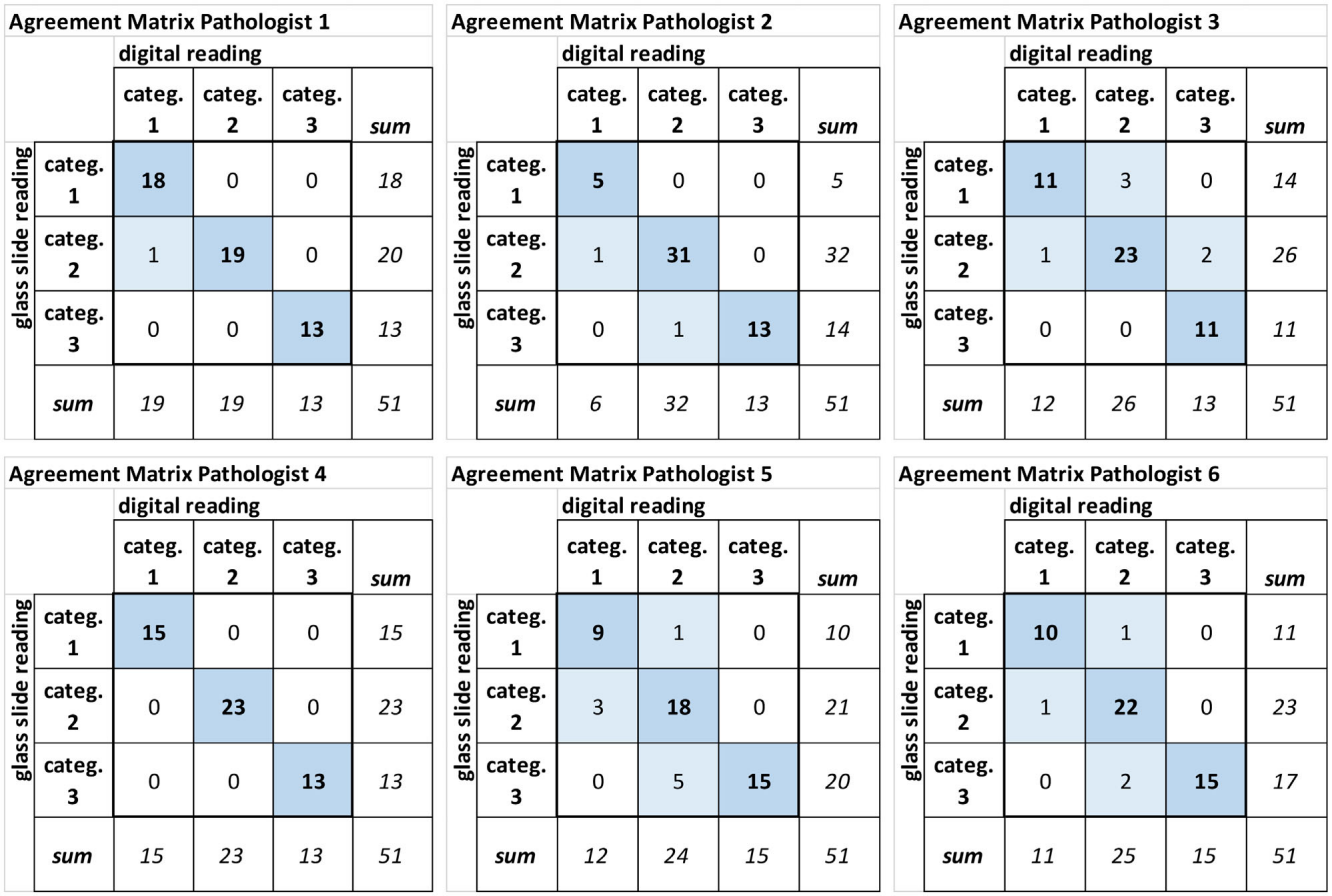
Pathologist's agreement matrices of PD‐L1 TPS categorical results for glass slide versus WSI reading. Each of the six agreement matrices shows the number of cases assigned by a specific participating pathologist to category 1 (PD‐L1 TPS <1%), category 2 (1% ≤ PD‐L1 TPS < 50%), and category 3 (PD‐L1 TPS ≥50%) for glass slide versus digital (WSI) reading. [Color figure can be viewed at wileyonlinelibrary.com]

The PD‐L1 TPS percentage results by glass slide reading versus WSI (Figure 2) reading and the agreement matrices according to the TPS categories for each of the six participating pathologists are presented in Figures 3 and 4, respectively.

Overall agreement and specific agreement for each category were calculated based on the agreement matrices. For the six participating pathologists, the overall intraobserver agreement was in the range of 88%–100%, specific intraobserver agreement for category 1 (PD‐L1 TPS <1%) 82%–100%, for category 2 (1% ≤ PD‐L1 TPS < 50%) in the range of 80%–100%, and for category 3 (PD‐L1 TPS ≥50%) in the range of 86% to 100% (Table S1).

ICC values (at the lower bound of the 95% CI) for all six pathologists, for the estimation of intraobserver agreement based on the PD‐L1 TPS percentage results, ranged from 0.911 to 0.993 (excellent agreement) (Table S2).

Cohen's kappa values for intraobserver agreement of six pathologists based on the PD‐L1 TPS categorial results ranged from 0.536 to 1.000 (Table S3).

### Interobserver agreement for PD‐L1 TPS scoring between pathologists

For the glass slide reading results of the six pathologists, we found an ICC of 0.915 (excellent agreement, 95% CI: 0.873–0.947). For the WSI reading results of the six pathologists, the ICC was 0.953 (excellent agreement, 95% CI: 0.931–0.971).

No substantial differences in the interobserver agreement of the six participating pathologists' PD‐L1 TPS categorical results by glass slide and WSI reading were observed (Table 1). Fleiss' kappa for WSI reading were in the range of 0.558 (moderate agreement) for category 1 (PD‐L1 TPS <1%) to 0.873 (almost perfect agreement) for category 3 (PD‐L1 TPS ≥50%). For glass slides reading, Fleiss's kappa was in the range of 0.541 (moderate agreement) for category 2 (1% ≤ PD L1 TPS < 50%) to 0.796 (substantial agreement) for category 3 (PD L1 TPS ≥50%).

**Table 1:** Fleiss’ kappa as a measure of interobserver agreement of the six participating pathologists' PD‐L1 TPS categorical results by WSI reading and by glass slide reading

	WSI reading Fleiss kappa (95% CI)	Glass slide reading Fleiss‐kappa (95% CI)
Overall agreement (all categories)	0.662 (0.611–0.713)	0.640 (0.589–0.691)
Specific agreement for category 1 (PD‐L1 TPS <1%)	0.558 (0.488–0.629)	0.601 (0.530–0.671)
Specific agreement for category 2 (1% ≤ PD‐L1 TPS < 50%)	0.574 (0.503–0.644)	0.541 (0.470–0.612)
Specific agreement for category 3 (PD‐L1 TPS ≥50%)	0.873 (0.803–0.803)	0.796 (0.725–0.867)

### Interobserver agreement for PD‐L1 TPS scoring between pathologists and AI

As a measure of interobserver agreement between pathologists and algorithms, we calculated Fleiss' kappa between the median PD‐L1 TPS result of the six participating pathologists' WSI readings and the PD‐L1 TPS result of the algorithm for categorical data (Table 2).

**Table 2:** Fleiss' kappa as a measure of interobserver agreement between the median PD L1 TPS result of the six participating pathologists' WSI readings and the PD‐L1 TPS result of automatic PD‐L1 analysis algorithms A1 and A2 for categorical data

	Fleiss kappa (asymptotic 95% CI)			
	Overall agreement for all categories	Specific agreement for category 1 (PD‐L1 TPS <1%)	Specific agreement for category 2 (1% ≤ PD‐L1 TPS < 50%)	Specific agreement for category 3 (PD‐L1 TPS ≥50%)
Six pathologists' interobserver agreement for WSI reading	0.662 (0.611–0.713)	0.558 (0.488–0.629)	0.574 (0.503–0.644)	0.873 (0.803–0.944)
A1 compared with median result of six pathologists for WSI reading	0.160 (−0.052 to 0.371)	0.183 (−0.103 to 0.468)	0.092 (−0.194 to 0.377)	0.247 (−0.039 to 0.533)
A2 compared with median result of six pathologists for WSI reading	0.551 (0.344–0.759)	0.536 (0.253–0.819)	0.540 (0.257–0.823)	0.590 (0.307–0.873)

**Figure 5:**
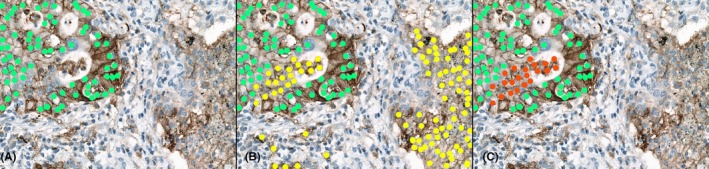
Illustration of potential pitfalls in pathologist's versus algorithmic evaluation of PD‐L1 expression. (**A**) Pathologists: Viable PD‐L1‐positive tumour cells are accurately identified (green dots), TPS = 75%. (**B**) A1: In addition to the viable PD‐L1‐positive tumour cells (green dots), macrophages (right yellow dots) and necrotic tumour cells (left yellow dots) are incorrectly counted as PD‐L1‐positive tumour cells, TPS = 45%. (**C**) A2: While macrophages are correctly excluded and not mistaken for tumour cells, necrotic tumour cells (red dots) are still erroneously identified as viable PD‐L1‐positive tumour cells (green dots), TPS = 60%. [Color figure can be viewed at wileyonlinelibrary.com]

We analysed the overall agreement for all three categories. Interobserver agreement for WSI was 0.662 (substantial agreement), for six pathologists with almost perfect agreement for category 3 (0.873). This result was significantly better than the overall agreement between A1 or A2 and the median result of six pathologists for all categories ranging from 0.160 (slight agreement) to 0.551 (moderate agreement), respectively. Interestingly, moderate agreement for all three specific categories was relatively stable for A2, while it was variable for A1, ranging from 0.092 (slight agreement) for category 2, to 0.247 (fair agreement) for category 3. Additionally, both algorithms exhibit a potential inability to confidently distinguish viable tumour cells from macrophages and necrotic tumour cells (Figure 5).

When comparing each pathologist's results to the median results of the other five pathologists at the PD‐L1 50% cutoff, Fleiss' kappa was in the range of 0.794 (95% CI lower bound 0.519) to 1.000 (95% CI lower bound 0.726) (Table S4).

## Discussion

The aim of our study was to evaluate the agreement of PD‐L1 scoring in NSCLC between pathologists and algorithms for PD‐L1 analysis. Our results demonstrated excellent intra‐ and interobserver agreement for pathologists. On the other hand, the agreement of pathologists´ score with two different, commercially available algorithms was lower than expected.

We are witnessing tremendous developments in computational pathology and their implementation into clinical practice. The FDA approved WSI usage for primary diagnostics in surgical pathology,^24^ and granted a 5 l0(k) clearance to an AI algorithm for detecting prostate cancer on WSI.^25^ Various algorithms were developed for the automatic evaluation of different (bio)markers and different diseases, which should provide help in diagnostic, prognostic, and predictive pathological evaluations in the near future.^26^ Recently, AI algorithms were also successfully utilized to discover new biomarkers and morphological features, for example, in lung or colorectal cancer.^27^, ^28^, ^29^

PD‐L1 TPS analysis is routinely done by pathologists using the visual estimation method and light microscopy. The TPS interpretation may be challenged by the presence of nontumour cells that also express PD‐L1, such as macrophages, necrotic cells, and immune cells. Visual assessment of PD‐L1 expression under the light microscope can be time‐consuming and can result in different PD‐L1 positivity estimations, particularly if a large number of cases need to be reviewed. It has been shown that this can be associated with increased interobserver variability.^6^, ^30^, ^31^ Therefore, the idea to have algorithms performing this scoring, eliminating human limitations, seems very appealing. Of note, in contrast to radiology, where AI commonly supports “concurrent” or “second readings”, in pathology AI holds potential as a first‐line tool for initial screening and quantification. Studies suggest that AI‐augmented pathology can enhance diagnostic sensitivity and specificity across various histologic grades and tumour sizes, supporting its viability as a first‐reading tool. Furthermore, as some countries face a shortage of pathologists, these solutions should save time, allowing additional focus on cases that cannot be resolved using AI.^6^, ^32^

Our study systematically evaluated intraobserver and interobserver agreement in PD‐L1 TPS scoring among pathologists using both glass slides and digital WSI. Overall intraobserver agreement was high, ranging from 88% to 100%, stressing the scoring consistency across mediums. The lowest intraobserver agreement was noted in category 2 (1% ≤ PD‐L1 TPS <50%).

The interobserver agreement, as indicated by ICC values, was excellent for both glass slide (0.915) and WSI readings (0.953). This high level of consistency among pathologists suggests the reliability of both traditional and digital slides in PD‐L1 TPS scoring. When evaluating the agreement between pathologists and AI algorithms, the results showed varying levels of concordance. The Fleiss' kappa values at the PD‐L1 TPS 50% cutoff point were 0.247 for algorithm A1 and 0.590 for algorithm A2, indicating fair to moderate interobserver agreement.

This poor performance of both algorithms tested in our study can be attributed to several factors. The most common problem for algorithms was misidentification of PD‐L1‐expressing macrophages and immune cells as tumour cells. Additionally, the identification and exclusion of necrotic tumour cells proved to be particularly challenging, as the algorithms frequently failed to exclude these cells from the analysis. Furthermore, the quality of staining, especially the contrast levels, had a significant impact on the performance of the AI systems. This was especially obvious by A1 approved for application as *in vitro* diagnostics only for a specific clone of PD‐L1 (SP263), used in a ready‐to‐use kit on a specific staining platform. In addition, the glass slides had to be scanned using a specific scanner. These technical variables introduced additional complexity, further complicating the process of automated assessment in everyday practice. For the A1 algorithm, tumour areas need to be manually marked on the WSI, while tumour area for A2 is fully automated and does not require manual annotation of tumour cells. Algorithm A2, on the other hand, is designated for research‐use‐only (RUO), and is independent of all previously mentioned requirements, and is able to identify the tumour area alone. Having this in mind, meticulous manual annotation for A1 could potentially enhance performance. However, the process, requiring detailed annotations across multiple fields and complex exclusion annotations, would be very time‐consuming, undermining AI's efficiency benefits. Moreover, definitive exclusion of all problematic cells may not always be feasible. Therefore, algorithms in DP should aim to minimize or eliminate the need for manual intervention, focusing on robust, accurate cell classification with minimal human input.

It is very interesting to compare our results with previously published studies. A recent study by van Eekelen *et al*.^18^ showed similar results with our findings, with moderate interobserver agreement (0.68) and AI performance slightly below the pathologist benchmark (0.55), and as in our study, variability in performance across different expression levels. On the other hand, some studies have shown promising results with AI‐based PD‐L1 scoring. Kim *et al*.^32^ reported concordance of TPS between AI and pathologists according to TPS ≥50%, 1%–49%, and <1% of 85.7%, 89.3%, and 52.4%, respectively, while Widmaier *et al*.^33^ demonstrated high correlation coefficients (Spearman and Pearson ranging from 0.83 to 0.95) between pathologist evaluations and automated algorithms. Another study by Haragan *et al*.^34^ demonstrated a strong concordance of the Roche uPath model with the manual scoring by pathologists, but the most important aspect was that the interpathologist concordance was significantly improved when the AI model was used, i.e. Fleiss' kappa increased from 0.613 to 0.886, and the ICC improved from 0.837 to 0.954.

A very important aspect, that was not investigated in our study, is also the clinical validation; in other words, how obtained scores relate to therapy response. Cortellini *et al*.^35^ using the same AI model (uPath—Roche) demonstrated clinical relevance in a series of patients with advanced‐stage NSCLC previously treated with a single agent PD‐1/PD‐L1 checkpoint blockade. The uPath PD‐L1 score demonstrated predictive ability for risk of death with an AUC of 0.81 (95% CI: 0.66–0.91), and response rate of uPath high vs. low of 51.6% and 25.0%, respectively.

There are several limitations of our study. The sample size, as a major limitation of this study, was limited by the availability of archival tissue samples and the feasibility of conducting foreseen analysis within the timeframe of the study. A larger number of study cases would be needed to further confirm our observations. Furthermore, all samples were retrospective and originated from a single institution. It would be essential to perform a larger prospective multi‐institutional study to further evaluate the performance of the two algorithms used in our study. It remains uncertain if different tissue processing protocols would result in a different performance.

Despite the potential of AI algorithms in automating PD‐L1 scoring, our study highlights significant limitations in their current application. Notably, these algorithms demonstrate a surprising inability to confidently distinguish viable tumour cells from macrophages and necrotic tumour cells. This deficiency means that AI does not yet match the consistency and reliability of expert pathologists, particularly at clinically significant TPS cutoffs of 1% and 50%. Such findings underscore the imperative for ongoing refinement and rigorous validation of AI tools. A critical and meticulous approach to their development will be beneficial in the long run, enhancing their accuracy and reliability. Only through such refinements can AI effectively augment pathologists' capabilities, ultimately improving diagnostic workflows and patient outcomes by reducing assessment time and minimizing variability in PD‐L1 scoring.

## Author contributions

M.P., G.E.O., M.K., and L.B. performed study concept and design; M.Z., C.M., and L.B gathered the cohort; M.P. L.K., and L.B. performed development of methodology; S.D, I.K., M.Z., H.P., J.F., S.I., and L.B. performed slide evaluation; M.P., G.E.O., M.K., and L.K. provided acquisition, analysis, and interpretation of data, and statistical analysis; M.P., G.E.O., S.D, I.K. M.K., and L.B. wrote the article. review and revision of the article; M.P., M.K., and C.M. provided technical and material support. All authors provided critical review and revision of the article. All authors read and approved the final article.

## Funding information

Parts of this work received funding from the European Union's Horizon Europe research and innovation program under no. 101079183 (BioMedAI TWINNING) and No. 101097036 (ONCOSCREEN). This publication reflects only the authors' view and the European Commission is not responsible for any use that may be made of the information it contains. Parts of this work received funding from the Austrian Science Fund (FWF), Project P‐32554 (Explainable Artificial Intelligence).

## Conflict of interest

L.B.—author received grants or contracts from Takeda, Roche, AstraZeneca, and BMS. The author received payment or honoraria for lectures, presentations, speakers' bureaus, article writing or educational events from Invitae, Eli‐Lilly, AstraZeneca, Roche, MSD, Merck, BMS, Pfizer, Novartis, Takeda, Janssen, and Daiichi Sankyo. The author received support for attending meetings from Pfizer. The author participated in advisory boards of Invitae, Eli‐Lilly, AstraZeneca, Roche, MSD, Merck, BMS, Pfizer, Novartis, Takeda, and Janssen. The author is currently Int. Secretary‐Austrian Society of Pathology; member of PPS Membership, and Awards Committee; Member of the Rare Cancers Committee of the IASLC. The remaining authors have no conflict of interest to disclose.

## Statement on generative AI

During the preparation of this work the author(s) used ChatGPT (version 4‐o) to language check the article. After using this tool/service, the author(s) reviewed and edited the content as needed and take(s) full responsibility for the content of the publication.

## Supporting information


**Data S1.** Supplementary information.
**Table S1.** Raw intra‐observer agreement indices for pathologists' PD‐L1 TPS categorical results (category 1: PD‐L1 TPS <1%, category 2: 1% ≤ PD‐L1 TPS <50%, and category 3: PD‐L1 TPS ≥50%) for glass slide versus digital (WSI) reading.
**Table S2.** Intraclass Correlation Coefficient for pathologists' PD‐L1 TPS percentage results for glass slide versus WSI reading.
**Table S3.** Cohen's kappa as a measure of intra‐observer agreement of pathologists' PD‐L1 TPS scoring for glass slide and digital reading.
**Table S4.** Fleiss' kappa as a measure of inter‐observer agreement between the median PD‐L1 TPS result of the six participating pathologists' WSI readings and the PD‐L1 TPS result of automatic image analysis algorithms for dichotomous data at the TPS 50% cutoff.

## Data Availability

The data that support the findings of this study are available on request from the corresponding author, L.B. The data are not publicly available because it contains information that could compromise the privacy of research participants.
