# Supplementary material for: Comparative performance of PD‐L1 scoring by pathologists and AI algorithms
Source: Histopathology. 2025 Feb 17;87(1):90–100. doi: 10.1111/his.15432 (PMC12129605; doi:10.1111/his.15432)
Supplement: Supplementary file 1 — Data S1. Supplementary information. Table S1. Raw intra‐observer agreement indices for pathologists' PD‐L1 TPS categorical results (category 1: PD‐L1 TPS <1%, category 2: 1% ≤ PD‐L1 TPS <50%, and category 3: PD‐L1 TPS ≥50%) for glass slide versus digital (WSI) reading. Table S2. Intraclass Correlation Coefficient for pathologists' PD‐L1 TPS percentage results for glass slide versus WSI reading. Table S3. Cohen's kappa as a measure of intra‐observer agreement of pathologists' PD‐L1 TPS scoring for glass slide and digital reading. Table S4. Fleiss' kappa as a measure of inter‐observer agreement between the median PD‐L1 TPS result of the six participating pathologists' WSI readings and the PD‐L1 TPS result of automatic image analysis algorithms for dichotomous data at the TPS 50% cutoff. [file HIS-87-90-s001.docx]

**Supplemental Material**

*Table S1: Raw intra-observer agreement indices for pathologists’ PD-L1 TPS* ***categorical results*** *(category 1: PD-L1 TPS <1%, category 2: 1% ≤ PD-L1 TPS <50%, and category 3: PD-L1 TPS ≥50%) for glass slide versus digital (WSI) reading*

|  | Overall Agreement | Specific Agreement  for Category 1 (PD-L1 TPS <1%) | Specific Agreement  for Category 2 (1% ≤ PD-L1 TPS <50%) | Specific Agreement for Category 3 (PD-L1 TPS ≥50%) |
| --- | --- | --- | --- | --- |
| Pathologist 1 | 98% | 97% | 97% | 100% |
| Pathologist 2 | 96% | 91% | 97% | 96% |
| Pathologist 3 | 88% | 85% | 88% | 92% |
| Pathologist 4 | 100% | 100% | 100% | 100% |
| Pathologist 5 | 82% | 82% | 80% | 86% |
| Pathologist 6 | 92% | 91% | 92% | 94% |

*Table S2: Intraclass Correlation Coefficient for pathologists’ PD-L1 TPS percentage results for glass slide versus WSI reading*

|  | Intraclass Correlation Coefficient^a^ (95%CI) |
| --- | --- |
| Pathologist 1 | 0.981 (0.967-0.989) |
| Pathologist 2 | 0.981 (0.962-0.990) |
| Pathologist 3 | 0.950 (0.911-0.972) |
| Pathologist 4 | 0.987 (0.977-0.993) |
| Pathologist 5 | 0.959 (0.926-0.977) |
| Pathologist 6 | 0.976 (0.958-0.986) |

*(* ^a^ *two-way random effects model with measures of absolute agreement, single measurement)*

*Table S3:* ***Cohen’s Kappa*** *as a measure of intra-observer agreement of pathologists’ PD-L1 TPS scoring for glass slide and digital reading.*

|  | categorical data:  (PD-L1 TPS <1%),  (1% ≤ PD-L1 TPS <50%) (PD-L1 TPS ≥50%)) | | dichotomous data  at 1% cutoff:  (PD-L1 TPS <1%), (PD-L1 TPS ≥1%) | | dichotomous data  at 50% cutoff:  (PD-L1 TPS <50%), (PD-L1 TPS ≥50%) | |
| --- | --- | --- | --- | --- | --- | --- |
|  | Cohen’s Kappa | 95% confidence interval lower bound | Cohen’s Kappa | 95% confidence interval lower bound | Cohen’s Kappa | 95% confidence interval lower bound |
| Pathologist 1 | 0.970 | 0.905 | 0.958 | 0.850 | 1.000 | 1.000 |
| Pathologist 2 | 0.925 | 0.808 | 0.898 | 0.628 | 0.950 | 0.812 |
| Pathologist 3 | 0.810 | 0.639 | 0.794 | 0.563 | 0.891 | 0.710 |
| Pathologist 4 | 1.000 | 1.000 | 1.000 | 1.000 | 1.000 | 1.000 |
| Pathologist 5 | 0.726 | 0.536 | 0.769 | 0.505 | 0.785 | 0.582 |
| Pathologist 6 | 0.876 | 0.747 | 0.884 | 0.677 | 0.909 | 0.771 |

According to interpretation suggestions for the medical domain (McHugh 2012) these kappa values should be interpreted as “weak agreement” (0.40-0.59) to “perfect agreement”.

*Table S4: Fleiss’ Kappa as a measure of inter-observer agreement between the median PD-L1 TPS result of the six participating pathologists’ WSI readings and the PD-L1 TPS result of automatic image analysis algorithms* ***for dichotomous data at the TPS 50% cutoff***

|  | Fleiss’ Kappa | asymptotic 95% confidence interval |
| --- | --- | --- |
| algorithm 1 compared with median result of the 6 pathologists at TPS 50% cutoff | 0.354 | 0.068-0.640 |
| algorithm 2 compared with the median result of the 6 pathologists at TPS 50% cutoff | 0.672 | 0.389-0.955 |
| pathologist 1 compared with the median result of the other 5 pathologists at TPS 50% cutoff | 1.000 | 0.726-1.000 |
| pathologist 2 compared with the median result of the other 5 pathologists at TPS 50% cutoff | 0.794 | 0.519-1.000 |
| pathologist 3 compared with the median result of the other 5 pathologists at TPS 50% cutoff | 1.000 | 0.726-1.000 |
| pathologist 4 compared with the median result of the other 5 pathologists at TPS 50% cutoff | 1.000 | 0.726-1.000 |
| pathologist 5 compared with the median result of the other 5 pathologists at TPS 50% cutoff | 0.902 | 0.627-1.000 |
| pathologist 6 compared with the median result of the other 5 pathologists at TPS 50% cutoff | 0.902 | 0.627-1.000 |
